# Needleless graft preparation for anterior cruciate ligament reconstruction with 4-strand semitendinosus autograft: a biomechanical in vitro study using a porcine model

**DOI:** 10.1186/s12891-024-08155-w

**Published:** 2024-12-16

**Authors:** Peter Melcher, Stefan Schleifenbaum, Yasmin Youssef, Philipp Rolzhäuser, Pierre Hepp, Jan Theopold

**Affiliations:** 1 Department of Orthopedics, Trauma and Plastic Surgery, University of Leipzig, Leipzig, Germany; 2 ZESBO - Center for Research On the Musculoskeletal System, University of Leipzig, Leipzig, Germany; 3 Faculty of Veterinary Medicine, Institute of Food Hygiene, University of Leipzig, Leipzig, Germany; 4 Department of Orthopedics and Trauma Surgery, Helios Klinik Leisnig, Colditzer Str. 48, Leisnig, 04703 Germany

**Keywords:** Anterior cruciate ligament, Reconstruction, SpeedTrap, Tensile forces

## Abstract

**Background:**

Ruptures of the anterior cruciate ligament (ACL) are common injuries. Reconstruction using autologous grafts is recommended to prevent further damage and functional impairment. Grafts are usually prepared with stabilizing sutures. The aim of this study was to evaluate if a 4-strand semitendinosus autograft preparation technique is non-inferior to conventional preparation techniques with regard to maximum tensile strength threshold.

**Methods:**

Fresh porcine flexor tendons were used as specimens in this study. Four different preparation techniques for quadruple-folded tendons were compared. Group 1 three suture FiberWire® (*n* = 20) and Group 2 one suture FiberWire® (*n* = 20) using Krakow stitches, Group 3 (*n* = 10) using SPEEDTRAP® and piercing the autograft and 4 (*n* = 9) using SPEEDTRAP® without piercing the autograft for preparation. Biomechanical tensile testing included 50 sinusoidal cycles of preloading between 50 and 150 N at 1 Hz and load-to-failure was measured at 20 mm/min.

**Results:**

Failure at the maximum load occurred at the filament for all samples, whereas failure of the suture/tendon interface was not observed. Load-to-failure was significantly higher in Group 1 (711 ± 91 N) than in all other groups. When comparing groups 2–4 load-to-failure was significantly higher in Group 2 (347 ± 24 N) than in Group 3 (258 ± 25 N, *p* < 0.02) but not than in Group 4 (325 ± 26N).

**Conclusion:**

In all 4 Groups the load to failure was higher than the maximum tension force on the construct that will be applied by hand (182N). Therefore, the needleless preparation technique seems to be a valuable alternative to conventional techniques for the insertion of the graft into the joint during joint-near tibial fixation technique.

## Introduction

Anterior cruciate ligament (ACL) ruptures are common injuries, particularly in young patients. The ACL is a central stabilizer of the knee joint, which prevents excessive anterior translation and rotational instability of the tibia relative to the femur. ACL reconstruction aims to restore the biomechanics of the knee to its pre-injury state, allowing for normal joint function and stability [1, 2]. While conservative treatment is possible, current research recommends the reconstruction of the ACL in young and active patients, as well as those with high knee instability or combined injuries to prevent instability, deteriorated kinematics, or (further) intra-articular damage [3, 4]. Sufficient and stable graft fixation is one of the predicting factors for successful ACL reconstruction, and initial stable fixation is essential to prevent elongation or failure before final engraftment [5]. Successful reconstruction depends on factors that include fixation strength, anatomical placement, and the mechanical properties of the graft [6].

Notably, different techniques have been described for ACL reconstruction, including the use of semitendinosus tendon allografts [1, 7, 8, 9]. Reconstruction options can be one-bundle or double bundle and anatomical and non-anatomical fixation. In one common technique a single-tendon technique is used, where the ACL graft is formed by a closed tendon loop secured with sutures on the ends of the graft [10]. Furthermore, different loops have been described, and most surgeons prepare their grafts by stabilizing sutures on each side of the graft. Biomechanical studies have demonstrated the effect of securing and strengthening the graft ends with sutures,, which is also referred to as tubularization. [11–13] Sufficient preparation of the graft ends is crucial as it can significantly impact the success of the reconstruction by ensuring optimal graft incorporation, stability, and overall knee function. Moralle et al. showed that tubularization of a quadruple-stranded hamstring graft positively impacts the mechanical properties of the graft [6]. However, tubularized grafts had a significantly higher yield load than those without tubularization [6]. In contrast, Wang et al. and Saur et al. showed that the piercing process of the tendon adversely affects its biomechanical properties as it leads to disruptions in the tendon fibers and creates soft-tissue risers [14, 15].

Recently, a new needleless preparation system for the tubularization of tendinous graft materials was introduced. The system allows for preparing the graft without piercing the soft tissue material, which as shown above can have an effect on the structural and biomechanical properties of the used graft. A current study has shown that graft preparation using the SpeedTrap system produces less structural damage to the allograft and is faster when compared to krackow stitches. In addition to that the SpeedTrap system showed higher strength, stiffness and energy at maximum load [16]. There however remains a limited number of biomechanical insights of the possible uses of the SpeedTrap system in comparison to other ACL preparation techniques. This study aims to compare the biomechanical strength of the standard SpeedTrap preparation technique and a fully needleless quadruple-folded semi-tendinosus grafts preparation technique for ACL reconstruction with two other conventional preparation methods.

## Methods

In this study, a biomechanical non-inferiority trial is conducted, comparing four different techniques using two different materials using a porcine model.

### Samples

Overall, 19 fresh porcine M. flexor digitorum specimens were used in this study. Tendons were harvested from the fresh forelegs of domestic pigs and cut to a length of 22 cm. Porcine flexor tendons were used for this experiment since their biomechanical properties are comparable to those of human semitendinosus tendons [17] and they have been used in previous studies with comparable test setups [18–21]. The tendons were frozen at −80 °C between harvest and biomechanical testing.

### Study set-up and Graft Preparation

**Fig. 1:**
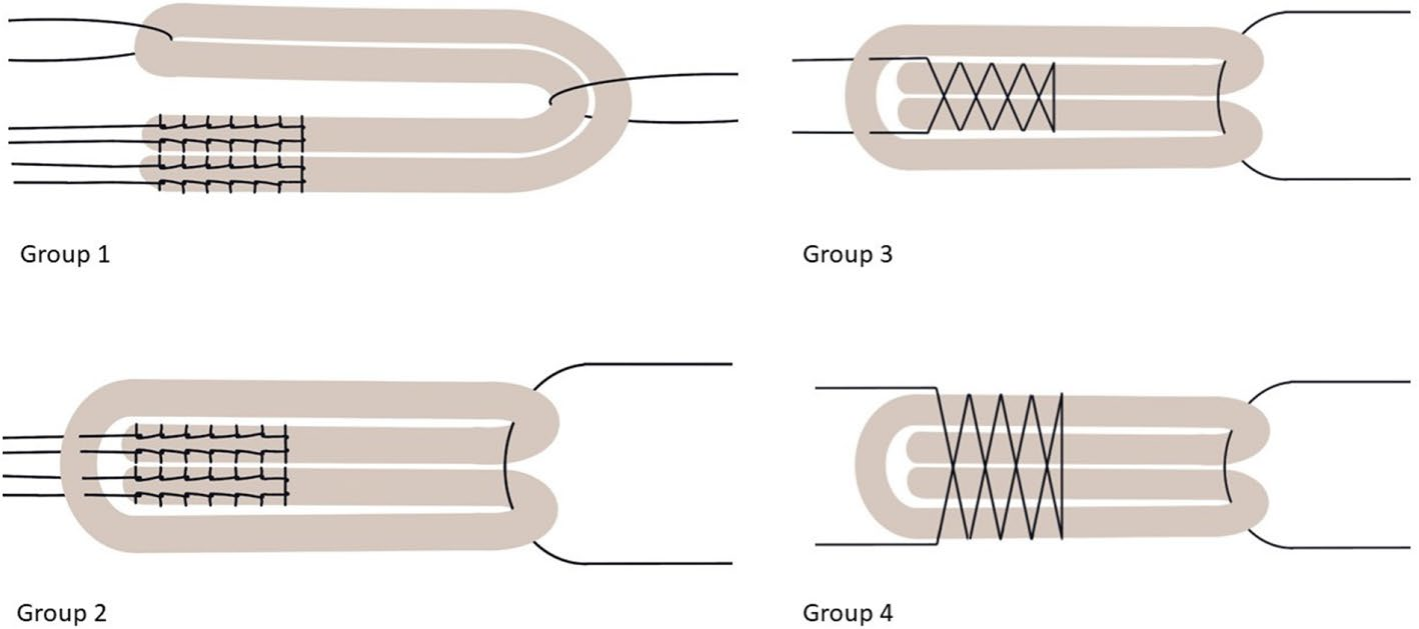
Preparation Techniques for quadruple folded allografts for ACL reconstruction: Group 1—classical FibreWire; Group 2—modified FibreWire; Group 3—classical SPEEDTRAP technique; Group 4 – modified needleless SPEEDTRAP technique

A non-inferiority study was performed to compare the standard SpeedTrap preparation with an adapted needleless SpeedTrap preparation and 2 other conventional preparation techniques for quadruple-folded ACL-reconstructions. Two experienced knee surgeons prepared the harvested tendon samples as quadruple-folded ACL grafts using SPEEDTRAP® (DePuy Synthes, Raynham, MA, USA). The samples were randomly assigned into two preparation groups (Group 3 and 4). The obtained data was compared to two preparation techniques previously utilized and applied by the authors (Groups 1 and 2) [18]. The different preparation techniques are graphically depicted in Fig. 1.

**Fig. 2:**
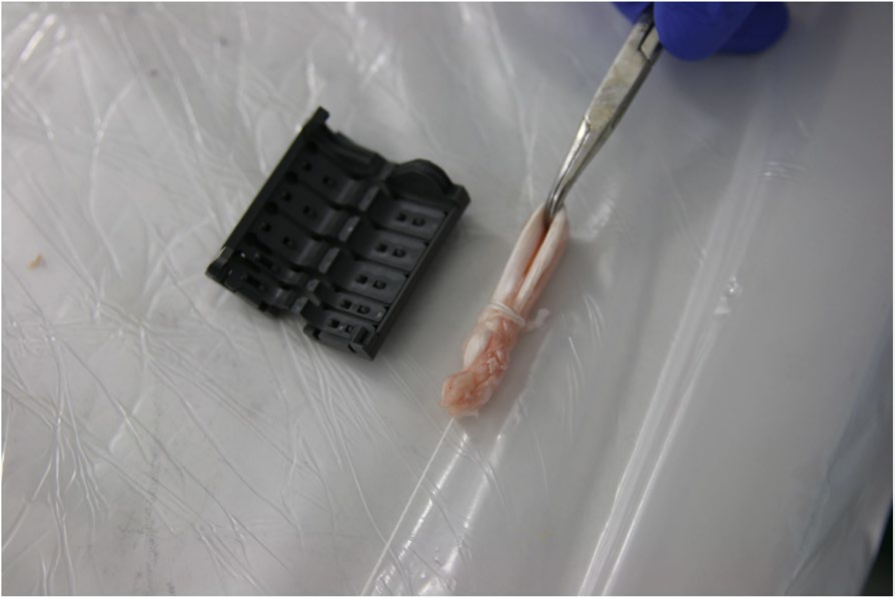
Preparation technique Group 4 – Modified SPEEDTRAP technique

1. Group 1 (*n* = 20): Classical FibreWire—Tendons were prepared using three tibial-sided sutures. The tendon ends were sutured separately using Krakow stitches with two FiberWire® #2 (Arthrex, Naples, FL, USA), and a third suture was looped around the midpoint of the tendon to form a four-stranded graft. (*n* = 20)
2. Group 2 (*n* = 20): FibreWire modified—The tendon ends were fixed using Krakow stitches and two FiberWire® #2 sutures. Both limbs of FiberWire® were stitched through the midpoint of the looped tendon to form a four-stranded graft.
3. Group 3 (*n* = 10): The classical SPEEDTRAP—A tendon loop was formed, and the tendon ends were prepared using SPEEDTRAP® (DePuy Synthes, Raynham, MA, USA). Both suture limbs were stitched through the midpoint of the looped tendon to form a four-stranded graft.
4. Group 4 (*n* = 9): Modified SPEEDTRAP–The tendon was folded in the middle twice to form a four-stranded graft, which was then fixed using SPEEDTRAP® (DePuy Synthes, Raynham, MA, USA) (Fig. 2).

### Mechanical testing

**Fig. 3:**
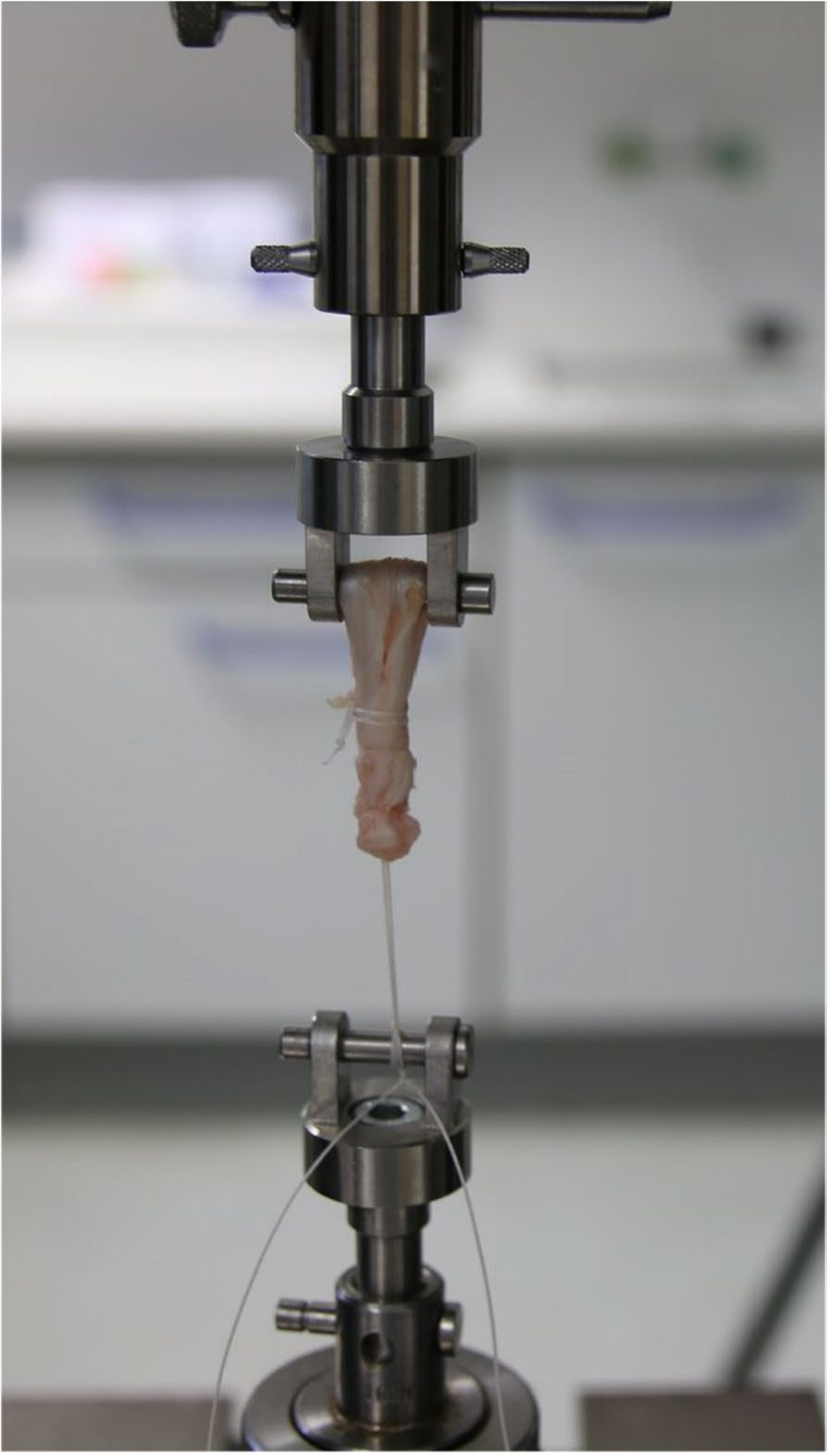
Test setup with prepared tendon fixed in the testing machine

The prepared graft samples were mounted into a mechanical setup on testing machine (Allround Line Z020, Zwick Roell GmbH, Ulm, Germany) with a hook and customized hold fastener to assess maximum load to failure in axial traction (Fig. 3). First 50 sinusoidal cycles of preloading between 50 and 150 N at 1 Hz were performed, and the load-to-failure was measured at 20 mm/min. In addition to that the mode of failure (suture failure / tendon-suture interface failure) was assessed as secondary outcome.

### Statistical analysis

Data were collected in Excel 2021 (Microsoft Corporation, Redmond, WA, USA) for the descriptive analyses. Statistical analysis was performed using the Statistical Package for Social Science (SPSS) V28.0 (IBM, Armonk, NY, USA). Univariate analysis and Tukey-Honestly Significant Difference (HSD) correction was conducted to compare the mean differences among the four groups. The level of statistical significance was set at *p* < 0.05.

## Results

**Fig. 4:**
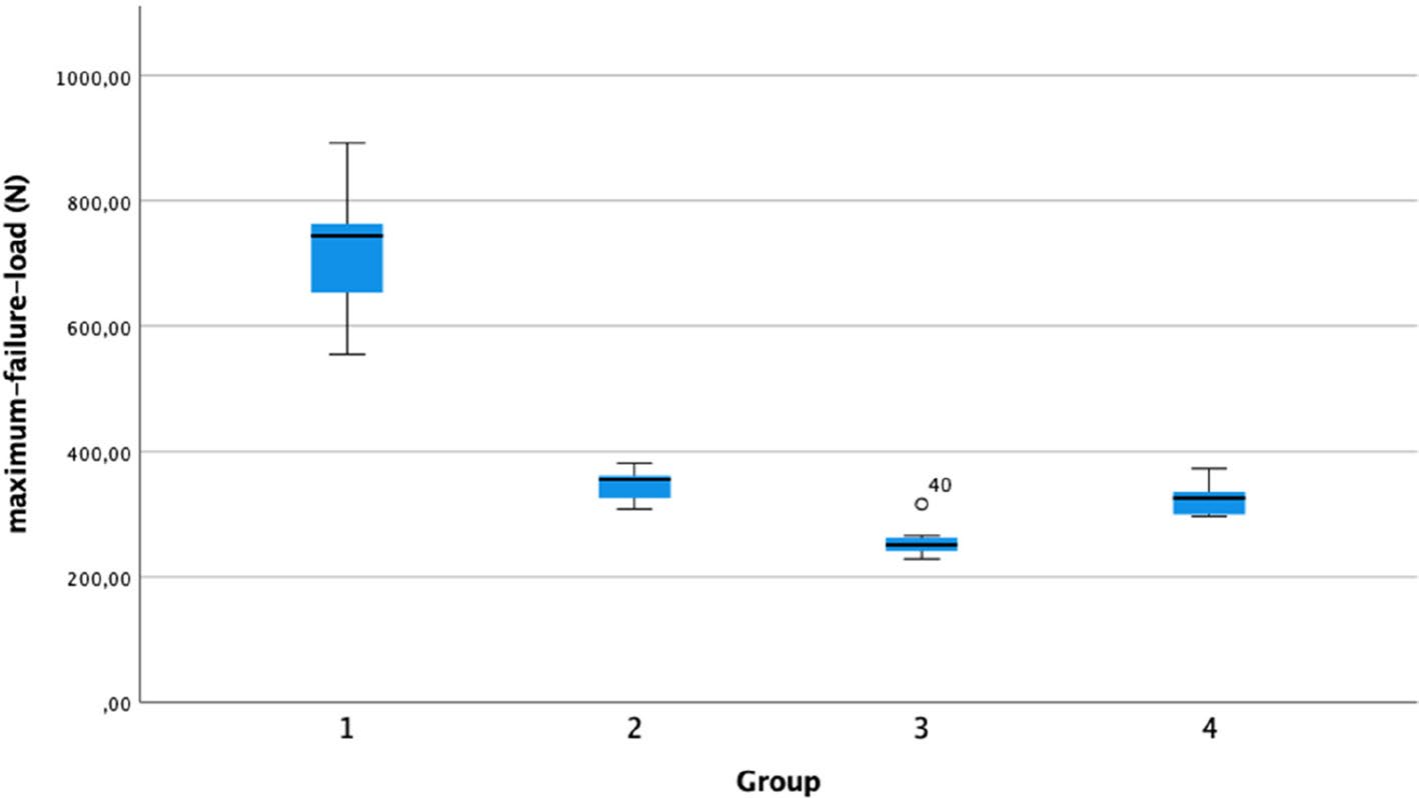
Boxplot graph showing average maximum failure load in Newton

Mechanical testing was possible in 56 of the 59 samples. One sample each from Groups 1 and 4 was excluded from testing due to the pre-existing damage to the tendon. However, one sample from Group 3 was excluded due to insufficient fixation in the testing machine. Consequently, 19, 20, 9, and 8 grafts were tested in Groups 1, 2, 3, and 4 respectively. In all samples, failure at the maximum load occurred at the filament. Failure at the suture/tendon interface was not observed. Load-to-failure was significantly higher in Group 1 (711 ± 91 N) than in all other groups. The load-to-failure was significantly higher in Group 2 (347 ± 24 N) than in Group 3 (258 ± 25 N, *p* < 0.02) but not in Group 4 (325 ± 26 N) (Fig. 4).

## Discussion

This study aimed to compare the biomechanical strength of a needleless ACL graft preparation technique with that of other conventional preparation methods. The results show that needleless graft preparation technique is not inferior to using Krakow stitches for 4-strand semi-tendinosus reconstruction techniques.

Group 1 (classical FiberWire®) revealed the greatest load-to-failure. In this group also the highest amount of FiberWire® #2 is used (*n* = 3). In contrast, Group 2 (modified FiberWire®) using 2 FiberWire® #2 and Group 3 (classical SPEEDTRAP®) had similar preparation techniques, where the two tendon ends were prepared separately to form two limbs, which were subsequently stitched through the midpoint of the graft to form a four-stranded tabularized graft. However, the difference between both groups was that Group 2 had stitches through the limbs and 2 FiberWire® where used, whereas those in Group 3 were needleless an 1 SPEEDTRAP® was used. In addition, the two groups differed in the material used (FiberWire® vs. DYNACORD® as part of the SPEEDTRAP®). Furthermore, the study design could not differentiate whether the statistically significant difference in the maximum failure load was caused by the difference in material or preparation technique.

In contrast, Groups 3 and 4 (modified SPEEDTRAP®) differed in the maximum failure load despite in both groups only 1 SPEEDTRAP® was used for preparation. Notably, the material–tendon contact surface failure was not observed in any of the samples. In addition, the maximum load observed was close to the evaluated knot security and the maximum load for DYNACORD® and FiberWire®. In all cases, failure of the construct was defined as failure of the suture. Suture failure was previously reported by Hong et al. [11].

Previous studies have suggested that the true mean intraoperative load during tibial-sided tensioning of the graft does not exceed 90 N [22–24]. In addition, a previous study reported a mean maximum tensile force of 134 ± 28 N for tensioning ACL grafts, of which the limiting factor was finger pain when pulling the wires without supporting tools [18]. Therefore, not attaining the maximum load of the construct (the minimum strength was 258 ± 25 N in Group 3) during an operation raises the question of clinical relevance caused by the maximum load differences of the different graft preparation techniques and if the maximum failure load is relevant for the outcome.

Some of the limitations of this study include those inherent to a biomechanical in vitro study on porcine tendons and not using the human semitendinosus tendon. First, the influence of drying and autolysis of tendons during the harvesting procedure and storage on their biomechanical behavior remains unclear, which limits the application of our findings to living tissues [25]. However, Domnick et al. demonstrated that freshly frozen porcine flexor tendons represent an appropriate substitute for human semitendinosus tendons [17]. Second, only axial forces were applied, whereas shear and torque forces that may also occur during ACL graft tensioning were not considered. However, it should be noted that both porcine flexor tendons and the mechanical setup described in this study have been previously used in other investigations to test the strength of the suture-tendon constructs [11, 17, 18, 21].

The influence on the load to failure caused by different suture materials (FiberWire® vs. DYNACORD®) and different amount of suture in the different groups (Group 1 = 3 Sutures, Group 2 = 2 sutures, Group 3 and 4 = 1 suture) cannot be specified. Since all tests failed in the area of the suture, the material properties seem to have a decisive influence on the failure load and the results of this study.

In summary, needleless ACL graft preparation using the described technique appears to be sufficiently stable when considering the tensile forces that can be expected intraoperatively [15, 26, 27].

In addition, some surgeons have postulated that the time from graft harvest to implantation may be a risk factor for contamination and infection; therefore, a reduced graft preparation time could reduce the complications of surgical-site infections [28]. It has been demonstrated by another research group that the use of needleless preparation techniques can reduce the graft preparation time [16].

Whether the needleless preparation technique truly proves to be non-inferior to the suturing techniques will need to be demonstrated by clinical studies.

## Conclusions

No failures in the suture-tendon interface were observed, only suture failures occurred. It is therefore to be assumed that the material properties in this study have a greater influence on the failure load than the preparation technique. The failure loads were greater than the tension forces, which could be expected intraoperatively. The needleless preparation technique seems to be an alternative to conventional techniques for preparing a 4-stranded semitendinosus autograft during joint-near tibial fixation, there is currently no clear biomechanical advantage.

## Data Availability

The datasets used and/or analyzed during the current study available from the corresponding author on reasonable request.
